# Investigating the Impact of rTMS in Combination With Antidepressant Medications on Residual Symptoms in Acute Depression

**DOI:** 10.62641/aep.v53i3.1860

**Published:** 2025-05-05

**Authors:** Hong Dai, Conghao Sun, Jinfeng Fei, Baohua Song

**Affiliations:** ^1^Department of General Psychiatry, Huzhou Third Municipal Hospital, The Affiliated Hospital of Huzhou University, 313000 Huzhou, Zhejiang, China; ^2^Department of Psychogeriatrics, Huzhou Third Municipal Hospital, The Affiliated Hospital of Huzhou University, 313000 Huzhou, Zhejiang, China; ^3^Department of Psychosomatic Disorders, Huzhou Third Municipal Hospital, The Affiliated Hospital of Huzhou University, 313000 Huzhou, Zhejiang, China; ^4^Physical Therapy Center, Huzhou Third Municipal Hospital, The Affiliated Hospital of Huzhou University, 313000 Huzhou, Zhejiang, China

**Keywords:** depression, residual symptoms, antidepressants, repetitive transcranial magnetic stimulation

## Abstract

**Background:**

After several rounds of optimized pharmacotherapy, approximately one-third of patients with depression still exhibit residual symptoms (RS). While repetitive transcranial magnetic stimulation (rTMS) is an established non-invasive treatment for depression, its effectiveness in treating RS associated with depression remains unclear. This study investigated the effectiveness of different frequencies of repetitive transcranial magnetic stimulatio rTMS combined with antidepressant drugs in treating RS of acute depression.

**Methods:**

This retrospective study included 110 acute depression patients hospitalized in the Huzhou Third Municipal Hospital between April 2020 and April 2022. The clinical data were analyzed, and patients were divided into a control group (n = 31 cases), a low-frequency rTMS (LF-rTMS) group (n = 37 cases), and a high-frequency rTMS (HF-rTMS) group (n = 42 cases). The control group received antidepressant medicines, the LF-rTMS group was treated with LF-rTMS stimulation of the right dorsolateral prefrontal cortex (DLPFC) in addition to standard antidepressant medication, and the HF-rTMS group was given HF-rTMS stimulation of the left DLPFC. These treatment modalities were continued for four weeks. Additionally, the 16-item Quick Inventory of Depressive Symptomatology (QIDS-16), the 24-item Hamilton Depression Rating Scale (HAMD-24), and the number of RS were observed before and after treatment in the three groups, and the clinical effectiveness rates were monitored across these three experimental groups.

**Results:**

After treatment, the total QIDS-16 score, the number of RS, and the total HAMD-24 score were significantly decreased among the three groups compared to the before-treatment levels (*p* < 0.05). Both the LF-rTMS and HF-rTMS groups exhibited lower QIDS-16 scores, fewer RS, and lower HAMD-24 total scores than the control group (*p *< 0.05). Following treatment, all three groups demonstrated a significant decrease in the QIDS-16 sleep scores for sleep onset, nighttime sleep, early morning awakening, and sleep duration compared to pre-treatment levels (*p *< 0.05). Furthermore, the LF-rTMS group had lower post-treatment scores for sleep onset and nighttime sleep than the HF-rTMS group (*p *< 0.05). Conversely, the HF-rTMS group exhibited lower scores for early morning awakening and sleep duration than the LF-rTMS group (*p *< 0.05). Additionally, both the LF-rTMS and HF-rTMS groups showed higher clinical effectiveness rates than the control group (*p *< 0.05).

**Conclusion:**

Our findings showed that HF-rTMS targeting left DLPFC and LF-rTMS targeting right DLPFC could effectively alleviate clinical symptoms in patients with RS of acute depression, thereby increasing the efficacy rate of treatment. However, regarding the sleep disorder factors evaluated by the QIDS-16, there were differences in the emphasis of improvements between HF-rTMS targeting left DLPFC and LF-rTMS targeting right DLPFC.

## Introduction

Depression is a common mental health condition manifested as persistent low mood, high recurrence rates, high morbidity, disability, and increased risk of suicide [1]. The incidence of depression-related complications has been growing due to the fast-paced modern social life and elevated societal competition [2]. This condition causes persistent depressive mood and impaired cognitive capabilities, which, in severe cases, can result in self-harm and suicidal attempts [3]. The primary goal of treating depression is complete remission of symptoms and restoration of pre-illness functionality. However, despite several rounds of optimized drug treatments, about one-third of patients experience residual symptoms (RS) [4].

RS symptoms include incomplete remission of depressive and non-depressive mood symptoms, collectively called residual somatic symptoms (RSS). These symptoms increase the risk of depression recurrence and chronic disease progression and may eventually lead to treatment-resistant depression, significantly impairing cognitive and social functioning [5]. Treatment for RS involves antidepressant medications, psychotherapy, and physical therapies [6].

Repeat transcranial magnetic stimulation (rTMS), a non-invasive brain stimulation approach, has been used for treating depression, particularly in refractory cases. This approach works by modulating cortical excitability, influencing neural pathways associated with depressive symptoms, altering cerebral blood flow, regulating neurotransmitter metabolism, and facilitating dopamine release in the brain [7]. The therapeutic efficacy of this method depends on several parameters, such as stimulation site, magnetic field orientation, stimulation frequency, intensity, and duration [8]. A meta-analysis has reported that high-frequency rTMS (HF- rTMS) targeting the left dorsolateral prefrontal cortex is effective in alleviating chronic pain and associated depressive symptoms [9]. However, HF-rTMS stimulation increases discomfort and increases the risk of adverse reactions. Alternatively, low-frequency rTMS (LF-rTMS) targeting the right dorsolateral prefrontal cortex (DLPFC) has been suggested as a viable option for treating depression [10]. A meta-analysis conducted by Pan *et al*. [11] demonstrated that combining LF-rTMS with antidepressants significantly reduced depression scores, improved cognitive function, and decreased inflammatory markers in patients with post-stroke depression compared to standard antidepressant treatment alone.

Currently, the therapeutic efficacy of rTMS at varying frequencies in treating RS of depression remains unclear. Therefore, this study assessed the clinical effectiveness of rTMS at different frequencies combined with antidepressants for treating RS of acute depression.

## Methods

### Inclusion Criteria

The inclusion criteria for this study were as follows: The inpatients or outpatients, whose RS of acute depression was diagnosed by psychiatrists in the Huzhou Third Municipal Hospital following the diagnostic criteria for depressive episodes outlined in the 10th edition of the International Classification of Diseases (ICD-10) [12], aged 18 to 60 years, and both patients and their families voluntarily participated in the study and provided signed informed consent.

### Exclusion Criteria

Exclusion criteria were set as follows: schizophrenia and depressive episodes with alcohol and drug dependence; depression due to physical illness; neurodegenerative diseases or cerebrovascular diseases (excluded by Computed Tomography (CT) or Magnetic Resonance Imaging (MRI)); severe cardiac, liver, renal dysfunction and metabolic diseases; cases with severe suicidal tendencies; pregnant and nursing patients; patients with implanted metal or electronic instruments at stimulation site (such as electronic cochlear, pulse generator, pacemaker); patients with a history of seizures or family history of epilepsy; patients previously treated with modified electra convulsive therapy (MECT) and rTMS; those with bipolar depressive disorder; those not agree to participate in the study or unable to attend regular follow-up; withdrawal of informed consent; occurrence of severe complications during the course of treatment, demanding discontinuation of study or modification of treatment plan; suicide or accidental death of patients during treatment; and loss of follow-up.

### Grouping of the Study Participants

Based on the predetermined inclusion and exclusion criteria, this study recruited 110 acute depression patients admitted to Huzhou Third Municipal Hospital between April 2020 and April 2022. Clinical data of the patients were retrospectively analyzed, and patients were categorized into three groups: the control group (n = 31), the LF-rTMS group (n = 37), and the HF-rTMS group (n = 42). Furthermore, all patients underwent a one-week antidepressant drug washout before starting the treatment, and the treatment course lasted for 4 weeks. The study design received approval from the Ethics Committee of Huzhou Third Municipal Hospital (Ethics approval number: 2020-022), and informed consent was obtained from patients or their family members. The study design complied with the principles outlined in the Declaration of Helsinki.

### Treatment Procedures 

The control group patients received treatment with Selective Serotonin Reuptake Inhibitor (SSRI) and Serotonin and Noradrenalin Reuptake Inhibitors (SNRI) antidepressants, including Escitalopram (10~20 mg/d; H20193308, Zhejiang Huahai Pharmaceutical Co., Ltd., Linhai, China), Venlafaxine hydrochloride sustained-release capsule (75~225 mg/d; H20143052, Beijing Fuyuan Pharmaceutical Co., Ltd., Beijing, China), and Sertraline (50~100 mg/d; H20080141, Zhejiang Huahai Pharmaceutical Co., Ltd., Linhai, China), given continuously for 4 weeks. Patients suffer with sleep disorders were given short-term oral alprazolam tablets (0.4~0.8 g/d; H11020890, Beijing Yimin Pharmaceutical Co., Ltd., Beijing, China).

Furthermore, the HF-rTMS group received HF-rTMS stimulation in addition to the treatment given to the control group. HF-rTMS stimulation was applied to the left DLPFC at a frequency of 10 Hz and an intensity of 100% of the exercise threshold. Each session included 40 sequences, with 5 s of continuous stimulation per sequence and 20 s intervals between sequences, resulting in 2000 stimulation pulses per day. Treatments were administered five times a week over four weeks.

Similarly, the LF-rTMS group received low-frequency rTMS in addition to standard treatment given to the control group. LF-rTMS stimulation targeted the right DLPFC at a stimulation frequency of 1 Hz and an intensity of 100% of the exercise threshold. Each session comprised 40 sequences with 5 s of continuous stimulation per sequence and a 20 s interval between sequences, resulting in 2000 pulses per day. Treatments were administered five times a week for four weeks.

### Outcome Measures

The 16-item Quick Inventory of Depressive Symptomatology (QIDS-16), the 24-item Hamilton Depression Rating Scale (HAMD-24), and the number of RS were determined before and after treatment. The QIDS-16, developed by Rush *et al*. [13], comprises 16 items across 9 dimensions, such as sleep problems, appetite changes, weight changes, fatigue, self-evaluation, reduced interest, difficulty concentrating, slow or increase in exercise, and suicidal thoughts. Each item was rated on a 4-point score ranging from 0 to 3. Furthermore, the number of RS was also analyzed based on the QIDS-16 criteria [13, 14].

The HAMD-24 [15] includes several dimensions: anxiety/somatization (items 10, 11, 12, 15, 17, and 13), weight (item 16), cognitive impairment (items 2, 3, 9, 19, 20, and 21), daytime changes (item 18), delay (items 1, 7, 8, and 14), sleep disruptions (items 4, 5, and 6), despair (items 22, 23, and 24) and others. Moreover, items were divided into 3-level scoring (such as 4, 5, 6, 12, 13, 14, 16, 17, 18, 21) or 5-level scoring (e.g., 1, 2, 3, 7, 8, 9, 10, 11, 15, 19, 20, 22, 23, 24). Three-level scoring items were scored as 0 points (none), 1 point (mild and moderate), and 2 points (severe). Five-level scoring items were rated as 0 points (none), 1 point (mild), 2 points (moderate), 3 points (severe), and 4 points (extremely severe).

Clinical efficacy was determined based on the total score of QIDS-16. Obvious efficacy was defined as a reduction of >75% in the total QIDS-16 score compared to before treatment, effective as a decrease of 50%–75%, and ineffective as a decrease of <50%. The response rate was defined as the sum of the probabilities of patients categorized as either obviously effective or effective. Psychological assessments were performed by professionally trained medical staff.

### Statistical Analysis

Data were statistically analyzed using SPSS 23.0 software (IBM, Armonk, NY, USA). Shapiro-Wilk was used to test the normal distribution of the measurement data. Data following a normal distribution were presented as mean ± standard deviation ($\bar{x}$ ± s). Comparisons between groups were conducted using a one-way analysis of variance (ANOVA), the post-test method is “Tukey’s post-hoc test”, while within-group comparison was performed using a paired *t*-test. However, data not following a normal distribution were expressed as the median (interquartile range) [median with quartiles 1 and 3 (Q1, Q3)] and analyzed using a non-parametric test. Categorical data were expressed as number (n) and percentage (%), with the chi-square (χ^2^) test applied for comparisons among groups. The ranked data from independent samples were analyzed using the Kruskal-Wallis rank sum test. A *p*-value of <0.05 was considered statistically significant.

## Results

### Comparison of General Data Among the Three Groups

There were no significant differences among the three groups regarding gender, age, course of depression, body mass index (BMI), antidepressant drugs use and sedative hypnotics use (*p* > 0.05) (Table 1).

**Table 1.** **Comparison of baseline characteristics among the three groups [$\bar{x}$ ± s, n (%)]**.

Variables		Control group (n = 31)	LF-rTMS group (n = 37)	HF-rTMS group (n = 42)	*F/χ^2^*	*p*-value
Gender (Male/Female)		17 (54.84)/14 (45.16)	21 (56.76)/16 (43.24)	24 (57.14)/18 (42.86)	0.042	0.979
Age (years)		40.26 ± 7.47	38.73 ± 10.20	39.21 ± 8.93	0.250	0.780
The course of depression (months)		24.58 ± 8.98	26.65 ± 9.73	27.43 ± 9.21	0.857	0.427
BMI (kg/m^2^)		24.29 ± 2.87	24.62 ± 3.34	24.46 ± 2.90	0.098	0.907
Antidepressant drug use						
	Single drug	9 (29.03)	9 (24.32)	10 (23.81)	0.294	0.863
	≥2	22 (70.97)	28 (75.68)	32 (76.19)		
Sedative hypnotics use, yes		25 (80.65)	30 (81.08)	36 (85.71)	0.426	0.808

Note: LF-rTMS, low-frequency rTMS; HF-rTMS, high-frequency rTMS; BMI, body mass index.

### Comparison of the QIDS-16 Score Before and After Treatment Among the Three Groups

The QIDS-16 total scores showed no significant difference among the three groups before treatment (*p* > 0.05). However, a considerable reduction in QIDS-16 score was observed among these groups following treatment (*p* < 0.05). Additionally, the difference in the QIDS-16 scores between the LF-rTMS and HF-rTMS groups was comparable (*p* > 0.05). In contrast, both LF-rTMS and HF-rTMS groups had lower QIDS-16 scores than the control group (*p* < 0.05) (Table 2).

**Table 2.** **Comparison of the QIDS-16 score before and after treatment among the three groups [$\bar{x}$ ± s, score]**.

Experimental groups	n	Before treatment	After treatment	*t*	*p*-value
Control group	31	9.94 ± 4.55	6.03 ± 2.68	4.255	<0.001
LF-rTMS group	37	9.97 ± 4.27	4.35 ± 1.90^*^	7.101	<0.001
HF-rTMS group	42	11.14 ± 4.61	4.02 ± 1.81^*^	8.906	<0.001
*F*		0.912	8.768		
*p*		0.405	<0.001		

Note: QIDS-16, 16-item Quick Inventory of Depressive Symptomatology; LF-rTMS, low-frequency rTMS; HF-rTMS, high-frequency rTMS. After treatment, compared with the post-treatment control group, ^*^p < 0.05.

### Comparison of the Number of Residual Symptoms Before and After Treatment Among the Three Groups

Before treatment, the three experimental groups had no significant difference in RS (*p* > 0.05). However, after treatment, they showed a substantial reduction in symptoms (*p* < 0.05). Additionally, the LF-rTMS and HF-rTMS groups exhibited no significant difference in symptom reduction (*p* > 0.05), and both had fewer symptoms than the control group (*p* < 0.05) (Table 3).

**Table 3.** **Comparison of residual symptoms among the three groups before and after treatment [Median, (Q1, Q3)]**.

Experimental groups	n	Before treatment	After treatment	*Z*	*p*-value
Control group	31	7 (6, 9)	5 (4, 5)	4.562	<0.001
LF-rTMS group	37	8 (6, 10)	4 (2, 4)^*^	5.245	<0.001
HF-rTMS group	42	8 (6.75, 9)	3 (2, 4)^*^	5.443	<0.001
*Z*		0.969	14.614		
*p*		0.616	<0.001		

Note: Q1, quartile 1; Q3, quartile 3; LF-rTMS, low-frequency rTMS; HF-rTMS, high-frequency rTMS. After treatment, compared with the post-treatment control group, ^*^p < 0.05.

### Comparison of QIDS-16 Sleep Disorder Factors Before and After Treatment Among the Three Groups

The QIDS-16 scores for sleep disorder factors, including difficulty falling asleep, maintaining nighttime sleep, early morning awakenings, and total sleep duration, did not significantly differ among the three groups before treatment (*p* > 0.05). After treatment, the scores for difficulty falling asleep, nighttime sleep, early wake-up, and sleep duration were significantly lower across the three groups than those before treatment (*p* < 0.05). Additionally, post-treatment, the LF-rTMS group indicated substantially lower scores for falling asleep and nighttime sleep than the HF-rTMS and control groups (*p* < 0.05). In contrast, the HF-rTMS group had significantly lower scores for early morning awakenings and total sleep time compared to the LF-rTMS and control groups (*p* < 0.05) (Table 4).

**Table 4.** **Comparison of QIDS-16 sleep disorder factors before and after treatment among the three groups [Median (Q1, Q3), score]**.

Experimental groups	n	Time	Fall asleep	Night sleep	Early wake-up	Sleep time
Control group	31	Before treatment	2 (1, 3)	2 (1, 2)	2 (1, 2)	2 (1, 2)
		After treatment	1 (1, 1)^a^	1 (1, 2)^a^	1 (1, 2)^a^	1 (1, 2)^a^
LF-rTMS group	37	Before treatment	2 (2, 2)	2 (1, 2)	2 (1, 2)	2 (1, 2)
		After treatment	0 (0, 1)^a⁢b^	0 (0, 0)^a⁢b^	1 (0, 1)^a⁢b^	1 (0, 1)^a⁢b^
HF-rTMS group	42	Before treatment	2 (1, 2)	2 (1, 2)	2 (1, 2)	2 (1, 2)
		After treatment	0 (0, 1)^a⁢b⁢c^	1 (0, 1)^a⁢b⁢c^	0 (0, 1)^a⁢b⁢c^	0 (0, 0)^a⁢b⁢c^

Note: Q1, quartile 1; Q3, quartile 3; QIDS-16, 16-item Quick Inventory of Depressive Symptomatology; LF-rTMS, low-frequency rTMS; HF-rTMS, high-frequency rTMS. Within each group, the difference between post-treatment and pre-treatment was statistically significant, ^a^*p* < 0.05. After treatment, compared to the post-treatment control group, the difference was statistically significant, ^b^*p* < 0.05. After treatment, compared to the post-treatment LF-rTMS group, the difference was statistically significant, ^c^*p* < 0.05.

### Comparison of HAMD-24 Scores Before and After Treatment Among the Three Groups

No significant differences were observed in the HAMD-24 scores among the three groups before treatment (*p* > 0.05). However, after treatment, the HAMD-24 scores among all three groups were significantly reduced compared to before-treatment levels (*p* < 0.05). Additionally, the HAMD-24 scores were comparable between the LF-rTMS and HF-rTMS groups (*p* > 0.05), with both groups exhibiting lower scores than the control group (*p* < 0.05) (Table 5).

**Table 5.** **Comparison of the HAMD-24 scores before and after treatment among the three groups [Median (Q1, Q3), score]**.

Experimental groups	n	Before treatment	After treatment	*Z*	*p*-value
Control group	31	17 (14, 18)	8 (7, 10)	4.868	<0.001
LF-rTMS group	37	18 (15, 19)	7 (4, 8)^*^	5.308	<0.001
HF-rTMS group	42	16 (15, 18)	6 (4.75, 7.25)^*^	5.654	<0.001
*Z*		2.168	15.433		
*p*		0.338	<0.001		

Note: Q1, quartile 1; Q3, quartile 3; HAMD-24, 24-item Hamilton Depression Rating Scale; LF-rTMS, low-frequency rTMS; HF-rTMS, high-frequency rTMS. After treatment, compared with the post-treatment control group, ^*^p < 0.05.

### Comparison of Clinical Effective Rate Among the Three Groups

The comparison of the clinical effective rate between the LF-rTMS group and the HF-rTMS group revealed no statistical significance (*p* > 0.05). However, the LF-rTMS and HF-rTMS groups demonstrated a higher clinical effective rate than the control group (*p* < 0.05) (Table 6).

**Table 6.** **Comparison of clinical effective rate among the three groups [n (%)]**.

Experimental groups	n	Obvious efficacy	Effective	Ineffective	Total effective rate
Control group	31	8 (25.81)	8 (25.81)	15 (48.39)	16 (51.61)
LF-rTMS group	37	18 (48.65)	13 (35.14)	6 (16.22)	31 (83.78)^*^
HF-rTMS group	42	16 (38.10)	18 (42.86)	8 (19.05)	34 (80.95)^*^
χ ^2^					10.866
*p*					0.004

Note: LF-rTMS, low-frequency rTMS; HF-rTMS, high-frequency rTMS. Compared with the control group, ^*^p < 0.05.

## Discussion

The primary method of treating acute depression involves using antidepressant drugs, with the goal being clinical recovery—achieving complete symptom remission and functional recovery [16]. Although antidepressants provide a certain degree of therapeutic effect, their high cost and significant risk of side effects limit their widespread use [17]. Previous studies have shown that patients with acute depression often exhibit RS after treatment, such as sleep problems, persistent depression, loss of interest, fatigue, and anxiety, with sleep disturbances being the most common RS [18, 19]. Therefore, it is crucial to adopt effective methods to promote both symptom relief and functional recovery among RS patients.

Using antidepressants is a common method for treating RS in acute depression. RS is typically characterized by below-threshold depressive symptoms that persist after completion of treatment [20]. RS not only affects the quality of life of people with depression but also increases the risk of relapse [21]. In clinical practice, while antidepressant drugs may alleviate RS in some patients with insufficient treatment duration, the overall effect is often unsatisfactory, and substantial adverse effects can occur, resulting in poor treatment compliance [22, 23]. Meta-analyses have revealed that combining antidepressants with rTMS can improve clinical outcomes in treating depression [11, 24]. The widely accepted neuroplasticity hypothesis indicates that the central nervous system can adapt and change structurally and functionally in response to new stimuli experience and rTMS reduce depressive symptoms by targeting specific brain areas, such as long-term inhibition or improvement of neuronal activity, altering synaptic plasticity, and normalizing neuronal function [25, 26]. Our study revealed that both the LF-rTMS and HF-rTMS groups showed significant improvements in the QIDS-16 and HAMD-24 total scores, as well as a decrease in the number of RS, compared to the control group. Additionally, the overall effective rate in these groups was substantially higher than the control group. These findings indicate that combining rTMS with antidepressants is more efficacious than antidepressant medication alone in treating RS of acute depression.

The rTMS is a non-invasive therapeutic modality that uses magnetic fields to induce excitatory or inhibitory effects on cortical excitability beneath the stimulation coil by altering stimulus frequencies, thereby targeting specific brain regions [27, 28]. The cerebral hemispheres exhibit functional asymmetry in processes like emotional regulation, where greater activity in the left prefrontal cortex is associated with approach-related positive emotions, while greater activity in the right prefrontal cortex is linked to withdrawal-related negative emotions [29]. LF-rTMS inhibits cortical neuronal activity at the stimulation site, whereas HF-rTMS promotes cortical excitability [30].

Currently, sufficient evidence supports the antidepressant efficacy of HF-rTMS on the left DLPFC, which induces excitatory plasticity in areas of possible low activity, and the potential antidepressant efficacy of LF-rTMS on the right DLPFC, which induces inhibitory plasticity in areas of hyperactivity [31]. The findings indicated no statistically significant difference between LF-rTMS and HF-rTMS, combined with antidepressants, regarding the total QIDS-16 score, number of RS, the HAMD-24 scores, and overall clinical efficacy. These findings suggest that LF-rTMS and HF-rTMS offer comparable advantages in treating RS in acute depression. However, LF-rTMS and HF-rTMS showed different effects on common sleep-related RS. LF-rTMS indicated higher efficacy in improving the ability to fall asleep and increase nighttime sleep, while HF-rTMS was more effective in reducing early morning waking and elevating sleep duration. These variations may be due to the distinct mechanisms of each approach. LF-rTMS, as an inhibitory stimulus, likely alleviates cortical arousal cerebral and regulates melatonin secretion from the pineal gland, improving the ability to fall and stay at night [32, 33]. Conversely, HF-rTMS may enhance early waking by inducing and prolonging the transition from non-rapid eye movement to rapid eye movement sleep, thereby regulating circadian rhythms and improving overall sleep quality [34].

Our results provide valuable preliminary evidence for combining rTMS and antidepressants in treating RS of depression, laying the foundation for future large-scale, multi-center prospective studies. However, the limitations of this study may be as follows: (1) This single-center study included a limited number of patients. Larger, multi-center prospective studies are needed to validate these findings and implement their generalizability. (2) This study primarily relied on scale-based assessments, particularly the QIDS-16 scale, to evaluate improvement in depressive symptoms. Future studies must incorporate objective measures such as polysomnographic monitoring to offer a more comprehensive assessment of sleep quality and disorders [35]. This approach could deepen our understanding of the mechanisms by which HF-rTMS and LF-rTMS affect sleep disorders and help optimize treatment protocols. (3) This study only observed the effect of HF-rTMS and LF-rTMS in treating RS in acute depression. Additional investigation is warranted to assess whether combination or sequentially administering HF-rTMS and LF-rTMS could offer further therapeutic advantages.

## Conclusion

In summary, this study reveals that HF-rTMS targeting the left DLPFC and LF-rTMS targeting the right DLPFC can significantly ameliorate clinical symptoms in RS patients with acute depression and improve treatment efficiency. Additionally, the two methods exhibit distinct focuses on the improvement of sleep disorder factors as measured by the QIDS-16.

## Availability of Data and Materials

The data used to support the findings of this study are available from the corresponding author upon request.
